# Comparative analysis of blood and saliva expression profiles in chronic and refractory periodontitis patients

**DOI:** 10.1186/s12903-015-0150-3

**Published:** 2015-12-24

**Authors:** Bin Zhang, Ting Lin, Hong He

**Affiliations:** School and Hospital of Stomatology, Wuhan University, No.237 Luoyu Road, Hongshan District, Wuhan, 430079 China; Key Laboratory of Oral Medicine, Guangzhou Institute of Oral Disease, Stomatology Hospital of Guangzhou Medical University, Guangzhou, 510140 China

**Keywords:** Chronic periodontitis, Refractory periodontitis, Blood, Saliva, Functional and pathway enrichment analysis, MiRNA

## Abstract

**Background:**

This study aimed to identify characteristic representative genes through a comparative analysis of gene expression profiles in the blood and saliva of chronic periodontitis (CP) and refractory periodontitis (RP) patients to provide new treatment strategies that may be helpful in the treatment of different forms of periodontitis.

**Methods:**

GSE43525 was downloaded from Gene Expression Omnibus. In the dataset, thirteen samples were from blood including 4 controls, 4 CP and 5 RP samples, and ten samples were from saliva including 3 controls, 4 CP and 3 RP samples. After comparing the CP and RP samples, differentially expressed genes (DEGs) between these two types of periodontitis in the blood and saliva samples were identified by an LIMMA package. Then, functional and pathway enrichment analyses were performed by DAVID and KOBAS, respectively. The significantly associated miRNAs in CP and RP were searched by WebGestalt.

**Results:**

In total, 213 DEGs in CP and 45 DEGs in RP were identified. Functional enrichment showed that the DEGs of CP were mainly enriched in ribosome and regulation of apoptosis-related pathways in blood as well as saliva, while the DEGs of RP were significantly enriched in immune responses and response to organic substance-related pathways. Several miRNAs, such as miR-381 and miR-494, were identified as being closely associated with CP. In addition, *CD24*, *EST1*, *MTSS1*, *ING3*, *CCND2* and *SYNE2* might be potential targets for diagnosis and treatment of CP.

**Conclusion:**

The identified DEGs and miRNAs might be potential targets for the treatment of chronic and refractory periodontitis.

## Background

In recent years, periodontal diseases have become a major health problem worldwide. Periodontal diseases are infectious diseases caused by bacteria in the dental plaque [1]. Chronic periodontitis (CP) is an inflammatory destruction of tooth supporting structures that, if left untreated, may lead to tooth loss [2, 3]. CP affects nearly 50 % of the adult population and 60 % of the aged population globally [4]. CP is the most prevalent and destructive disease in adults [5]. In most periodontitis cases, therapeutic success is obtained for long periods of time [6]; however, a small proportion of treated patients, referred to as refractory periodontitis (RP) patients, do not respond well to properly performed conventional therapy and continue to show loss of periodontal attachment [7, 8]. The existence of specific pathogenic microbiotas may contribute to RP [9]. RP patients are very heterogeneous in terms of their clinical, microbiological and immunological characteristics [10].

CP is found in the presence of subgingival calculus and local factors [11]. The pathogenesis of CP is multifactorial in nature and results from interactions among bacterial, environmental, immunologic and genetic factors [12]. There is growing evidence that polymorphisms in interleukin 1 (IL1), IL6, IL10, etc. may be associated with CP in certain populations. Several cytokines, including tumour-necrosis factor-α (TNF-α), IL1, IL6, IL8, intercellular adhesion molecule-1 (ICAM-1), monocyte chemoattractant protein-1 (MCP-1), and granulocyte-macrophage colony-stimulating factor (GM-CSF), play important roles in the development process of periodontitis. The nuclear factor-κB (NF-κB) signalling pathway is involved in parts of the immune response, such as antigen presentation, immune balance and lymphocyte activation, and is significant in periodontitis [13]. CP is initiated and maintained in the first line by complex poly-microbial infection, and the innate susceptibility of the patient is now an accepted critical factor that will determine the destructive character of the disease [14]. An intraradicular infection persisting in the complex apical root canal system and extraradicular infection presenting in the form of periapical actinomycosis can lead to RP [15]. With high incidence and prevalent characteristics, the diagnosis and treatment of CP and RP has undoubtedly been a challenge for both clinical investigators and clinicians.

The aim of present study was to compare and analyse these two types of periodontitis at the genetic level in blood and saliva samples and to explore the possible mechanism of periodontitis. Biological microarray analysis was used to analyse the gene expression profile of CP and RP in different biological fluids with the aim of providing additional treatments and the ability to better distinguish periodontitis.

## Methods

### Microarray data

**Fig. 1:**
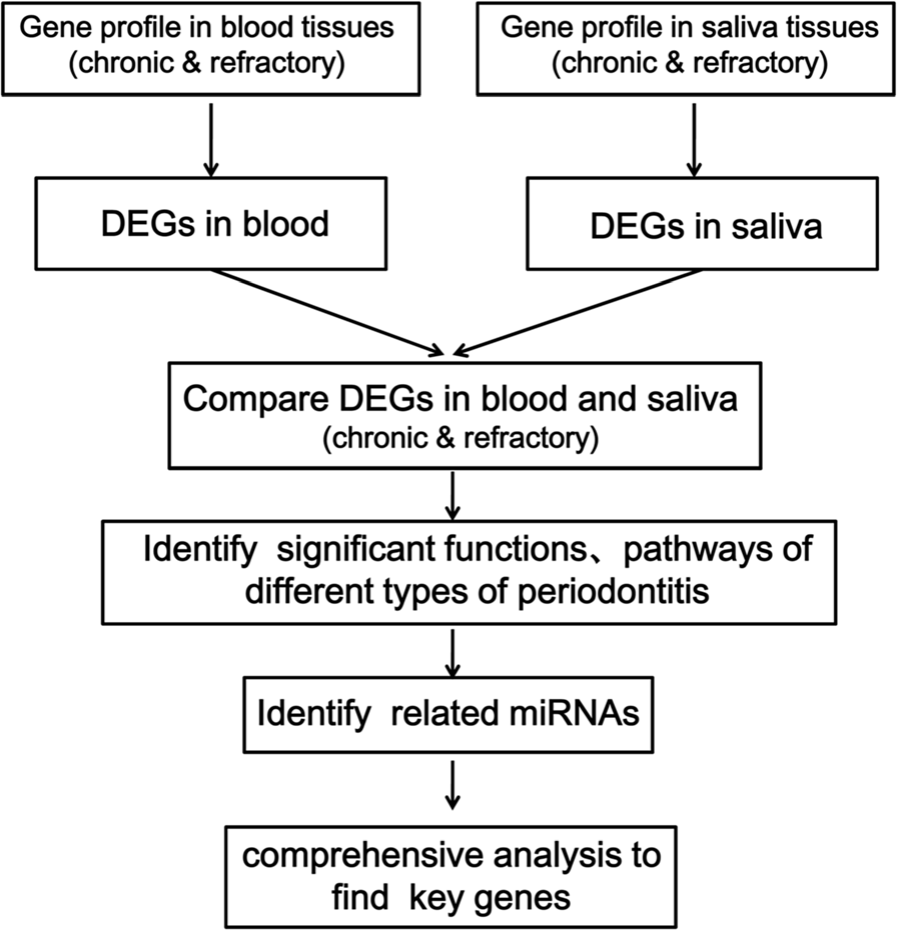
The research analysis process of blood and saliva tissues

The research analysis process is shown in Fig. 1 The gene expression profile of GSE43525 [16] was downloaded from Gene Expression Omnibus (GEO), which was based on the platform of the HumanHT-12 V4.0 expression beadchip. Each set of expression profiling data, which is submitted to the GEO database mainly by laboratories all around the world, includes platform information, series information and sample information [17]. A total of 13 samples (including 4 control samples, 4 CP samples and 5 RP samples) were obtained from the blood, and 10 samples (including 3 control samples, 4 CP samples and 3 RP samples) were from the saliva according to the website information (http://www.ncbi.nlm.nih.gov/geo/query/acc.cgi?acc=GSE43525). These samples were chosen from patients of different sexes and ages. All of the patients were from the Faculty of Dentistry, University of Toronto, Toronto, ON and provided informed consent. In additon, GSE43525 was deposited by Lakschevitz et al. whose study was approved by the Office of Research Ethics (ORE) at the University of Toronto (Protocol # 25698) [16].

### Comparative analysis of chronic and refractory periodontitis samples from blood and saliva

In accordance with the descriptions and characteristics of the original samples, the blood and saliva samples from the CP and RP patients were used for gene expression profile analysis.

### Data preprocessing and identification of differentially expressed genes (DEGs)

The probe-level data were converted into the expression measures. For each sample, the expression values of all probes for a given gene were reduced to a single value by taking the average expression value and then log2-transformation was conducted [18]. The linear model of microarray data (LIMMA) package in R [19] was used to identify the DEGs of CP and RP in the blood and saliva compared with normal controls. The *p*-value < 0.05 and |log fold change (FC)| > 1 were used as the cut-off criteria.

### Functional enrichment analysis of DEGs

Gene enrichment analysis is an analytical strategy using a set of similar or related genes as a whole by calculating the overall significance of changes in gene expression to identify whether the biological function or properties change. This strategy could greatly reduce the dimension of data analysis and makes the analysis process closer to biological problems, therefore this method is widely used in microarray technology [20]. Currently, there are many tools that can provide enrichment analysis of gene function. Among them, Database for Annotation, Visualization and Integrated Discovery (DAVID) is the most popular [21]. DAVID provides a comprehensive set of functional annotation tools for investigators to understand the biological meaning behind a large list of genes. We performed a Gene Ontology (GO) enrichment analysis in terms of biological progress (BP). The false discovery rate (FDR) < 0.05 was used as the cut-off criterion.

### Pathway analysis of DEGs

To better understand and further study the biological functions, the KEGG Orthology Based Annotation System (KOBAS) [22] was used to conduct the pathway annotation and enrichment analysis based on a hypergeometric distribution. The *p*-value < 0.05 was used as the cut-off criterion.

### Searching for significantly associated microRNAs

MicroRNA (miRNA) broadly participate in many biological processes and can be used as a biomarker for the diagnosis of a variety of diseases [23, 24]. We used the WEB-based Gene Set Analysis Toolkit (WebGestalt) to calculate the association between DEGs and miRNAs in CP and RP [25, 26]. The *p*-value < 0.05 was used as the cut-off criterion.

## Results

### Identification of DEGs

**Fig. 2:**
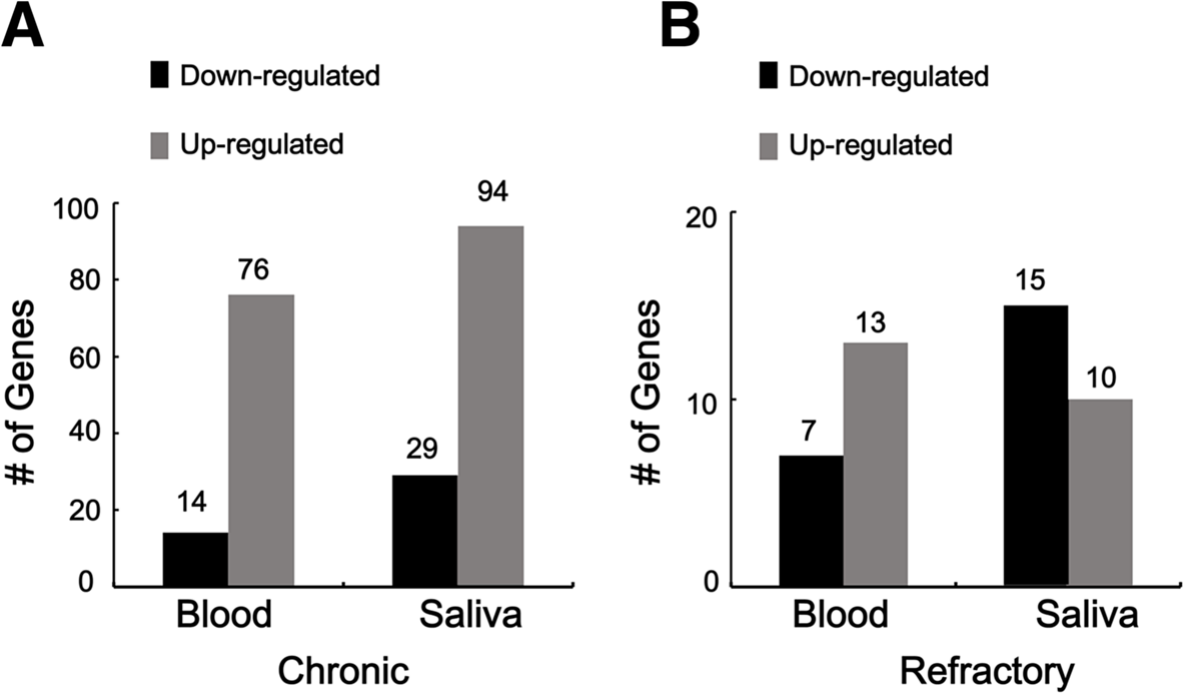
**a**: Up-regulated and down-regulated genes in the blood and saliva of patients with chronic periodontitis; **b**: Up-regulated and down-regulated genes in the blood and saliva of patients with refractory periodontitis. *black colour*: down-regulated genes; *grey colour*: up-regulated genes

After preprocessing the microarray data, the LIMMA package was used to screen the DEGs. A total of 213 DEGs in CP (including 76 up-regulated and 14 down-regulated genes in the blood as well as 94 up-regulated and 29 down-regulated genes in the saliva) and 45 DEGs in RP (including 13 up-regulated and 7 down-regulated genes in the blood, as well as 10 up-regulated and 15 down-regulated genes in the saliva) were identified (Fig. 2).

### Functional enrichment analysis of DEGs

To study the functions of DEGs in CP and RP in blood and saliva, the functional enrichment analysis of all these DEGs were performed by DAVID. The results showed that DEGs in the blood of CP patients were involved in translation-related BP terms (Table 1a). Meanwhile, the DEGs in the blood of RP patients were mainly enriched in immune response processes (Table 1b). Moreover, the DEGs in the saliva of CP patients were mainly involved in the regulation of apoptosis and cell death (Table 1c). In addition, the DEGs in the saliva of RP patients were mainly enriched in genes responsive to organic substances and bacterium (Table 1d).

**Table 1:** Gene Ontology enrichment analysis of differentially expressed genes of chronic and refractory in blood and saliva

a) Blood-chronic		
Term	Count	*P*-value
GO:0006414 ~ translational elongation	11	8.07E-11
GO:0006412 ~ translation	13	9.52E-08
GO:0006968 ~ cellular defense response	6	1.42E-05
b) Blood-refractory		
Term	Count	*P*-value
GO:0006955 ~ immune response	4	3.16E-02
c) Saliva-chronic		
Term	Count	*P*-value
GO:0042981 ~ regulation of apoptosis	12	2.64E-03
GO:0043067 ~ regulation of programmed cell death	12	2.85E-03
GO:0010941 ~ regulation of cell death	12	2.93E-03
GO:0008202 ~ steroid metabolic process	6	3.90E-03
d) Saliva-refractory		
Term	Count	*P*-value
GO:0010033 ~ response to organic substance	5	8.71E-03
GO:0009617 ~ response to bacterium	3	2.13E-02
GO:0019439 ~ aromatic compound catabolic process	2	2.34E-02
GO:0008286 ~ insulin receptor signaling pathway	2	4.29E-02
GO:0045787 ~ positive regulation of cell cycle	2	6.54E-02

### Pathway analysis of DEGs

To further understand the involved pathways of each set of DEGs, a pathway enrichment analysis was further performed. A total of 10 pathways were enriched for the DEGs (Table 2). The DEGs of the blood of CP patients were enriched in two pathways, the most significant being ribosome (*p* = 2.24E-10), which involved 11 DEGs. The DEGs in the blood of RP patients were mainly involved in five pathways, the most significant of which were cell adhesion molecules (CAMs). Meanwhile, the DEGs in the saliva of CP patients were enriched in the pathway of antigen processing and presentation. The DEGs in the saliva of RP patients were involved in tryptophan metabolism and aldosterone-regulated sodium reabsorption.

**Table 2:** The significanlty enriched pathways by differentially expressed genes

a) Blood-chronic	
Pathway	*P*-value
hsa03010:Ribosome	2.24E-10
hsa05332:Graft-versus-host disease	3.06E-02
b) Blood-refractory	
Pathway	*P*-value
hsa04514:Cell adhesion molecules (CAMs)	6.35E-03
hsa05330:Allograft rejection	3.49E-02
hsa05332:Graft-versus-host disease	3.78E-02
hsa04940:Type I diabetes mellitus	4.06E-02
hsa05320:Autoimmune thyroid disease	4.92E-02
c) Saliva-chronic	
Pathway	*P*-value
hsa04612:Antigen processing and presentation	1.24E-02
d) Saliva-refractory	
Pathway	*P*-value
hsa00380:Tryptophan metabolism	4.63E-02
hsa04960:Aldosterone-regulated sodium reabsorption	4.74E-02

### MiRNA-target gene analysis

WebGestalt was used to analyse the related miRNAs in CP and RP. Table 3 shows the relationship between miRNAs and DEGs. No target genes were found in the RP samples; however, there were 4 and 5 DEGs related to the miRNAs in the blood and saliva of CP patients, respectively. We found that cluster of differentiation 24 (*CD24*), esterase 1 (*EST1*), and metastasis suppressor 1 (*MTSS1*) were target genes for CP in blood, while, inhibitor of growth family member 3 (*ING3*), cyclin D2 (*CCND2*) and synaptic nuclear envelope protein 2 (*SYNE2*) were target genes for CP in saliva. The most significantly related miRNAs were miR-381 (which targets *ETS1* and *MTSS1*) and miR-494 (which targets *ING3*, *CCND2* and *SYNE2*).

**Table 3:** Target genes and their corresponding miRNAs in different groups

a) Blood chronic			
MiRNA	ID	*P*-value	Target genes
hsa_CTTGTAT,MIR-381	DB_ID:731	4.20E-02	ETS1, MTSS1
hsa_TGTGTGA,MIR-377	DB_ID:845	4.07E-02	CD24, ETS1
hsa_CAGCAGG,MIR-370	DB_ID:682	2.73E-02	SLAMF6, MTSS1
hsa_ATACTGT,MIR-144	DB_ID:702	4.47E-02	ETS1, ALDH1A3
b) Saliva chronic			
MiRNA	ID	*P*-value	Target genes
hsa_ATGTTTC,MIR-494	DB_ID:782	4.80E-03	ING3, CCND2, SYNE2
hsa_ACCATTT,MIR-522	DB_ID:749	4.80E-03	ING3, YWHAZ, CCND2
hsa_AGGTGCA,MIR-500	DB_ID:827	1.64E-02	CREB1, POFUT1
hsa_AGGGCAG,MIR-18A	DB_ID:668	3.35E-02	YWHAZ, CREB1
hsa_TAATGTG,MIR-323	DB_ID:724	4.38E-02	CREB1, TGFA

## Discussion

Periodontitis is a multifactorial infectious inflammatory condition characterized by a destructive inflammatory process affecting the supporting tissues of the tooth and resulting in alveolar bone resumption, periodontal pocket formation and eventually tooth loss [27]. CP is a typical disease that has a relatively mild phenotype and a slow progressing and chronic nature [28]. Functional enrichment showed that DEGs of RP were significantly enriched in immune responses. Additionally, persistent host inflammatory immune responses against pathogens results in the destruction of soft and mineralized periodontal tissues [29]. According to our results, functional level analysis showed that these two types of periodontitis could cause significant expression changes in the immune defence-related genes in the blood and saliva. As soon as the pathogens invade the periodontal tissue, the immune system uses a variety of methods including humeral immunity and cellular immunity to avoid the occurrence of periodontitis [30]. In our study, we obtained DEGs and their corresponding miRNAs in the blood and saliva of CP patients. Through searching for significantly associated microRNAs for the DEGs, the target genes including *CD24*, *EST1*, *MTSS1* and *ING3*, *CCND2*, and *SYNE2* were found. MiR-381 (which targets *ETS1* and *MTSS1*) and miR-494 (which targets *ING3*, *CCND2* and *SYNE2*) were the most significantly related miRNA in the blood and saliva. These miRNAs might function in CP by regulating their target genes.

*CD24* was found to be highly expressed in periodontitis. The consistent high expression of *CD24* has been reported in the epithelial attachment to the tooth and in the migrating epithelium of the periodontitis lesion, and titres of serum antibodies auto-reactive with *CD24* peptide correlates with the remission of periodontal disease [31]. Functional analysis revealed that *MTSS1*, which was initially described as a gene missing in invasive bladder cancer cell lines, was an actin binding protein involved in the regulation of actin cytoskeleton dynamics. *MTSS1* is shown to be sonic hedgehog (Shh) signaling dependent and synergize with the effects of Gli transcription factors [32]. *ING3* is a subunit of the nucleosome acetyltransferase of histone 4 (NuA4) complex, which activates gene expression. *ING3* is ubiquitously expressed in mammalian tissues and governs transcriptional regulation and cell cycle control [33]. Although reports on their roles in the progression of periodontitis are rare, we speculated that these genes might play key roles in periodontitis. *EST1*, *MTSS1*, *ING3*, *CCND2* and *SYNE2*, as well as their corresponding miRNAs, might be potential therapeutic targets for periodontitis. The serum antibody titres were increased in all patients with periodontitis to some degree [34]. The genetic susceptibility factors of periodontitis and quick identification of susceptible periodontitis patients will contribute to the prevention and treatment of periodontal disease.

## Conclusions

In summary, our data provides a comprehensive bioinformatics analysis of DEGs in the blood and saliva of CP and RP patients. A total of 213 DEGs in CP and 45 DEGs in RP were identified. *CD24*, *EST1*, *MTSS1*, *ING3*, *CCND2* and *SYNE2* may have the potential to be used as targets for periodontitis diagnosis and treatment.; however, more research is still needed to validate our findings.

## Abbreviations

BP: biological progress
CAMs: cell adhesion molecules
CP: chronic periodontitis
DEGs: differentially expressed genes
FC: fold change
FDR: false discovery rate
GEO: Gene Expression Omnibus
GO: Gene Ontology
KOBAS: KEGG Orthology Based Annotation System
NuA4: nucleosome acetyltransferase of histone 4
RP: refractory periodontitis
Shh: sonic hedgehog
